# Equine dystocia in Sweden (2006–2022): a retrospective analysis of mare and foal survival

**DOI:** 10.3389/fvets.2026.1910180

**Published:** 2026-09-04

**Authors:** Margo Verbruggen, Jane M. Morrell, Myrthe Wessel, Elin Olsson, Theodoros Ntallaris

**Affiliations:** 1 Arts and Dier, Bears, Netherlands; 2 Swedish University of Agricultural Sciences, Uppsala, Sweden; 3 Specialistische Voortplantingspraktijk, Amsterdam, Netherlands

**Keywords:** controlled vaginal delivery, equine obstetrics, fetotomy, foal survival, parturition, retrospective cohort study, time-to-resolution

## Abstract

**Background:**

Dystocia is an uncommon but potentially life-threatening complication of equine parturition. Due to early separation of the chorioallantois during the expulsive stage, fetal oxygen supply rapidly becomes compromised, making dystocia a time-critical emergency for both mare and foal. Objectives: To describe obstetrical interventions performed at equine referral hospitals, evaluate mare and foal survival, and identify factors associated with survival outcomes in a Swedish population where geographic distances may influence access to specialized veterinary care.

**Methods:**

Medical records from three equine referral hospitals in central Sweden were reviewed for dystocia cases presented between January 2006 and December 2022. Data collected included dystocia causes, obstetrical interventions, dystocia duration, prior veterinary intervention, and survival outcomes for mares and foals.

**Results:**

A total of 138 dystocia cases were included. Fetal causes of dystocia predominated (114/138), with malposition being most common (106/138). Controlled vaginal delivery was the most frequently performed intervention (33.3%, 46/138). Of 140 foals delivered, 17.1% were born alive and 12.1% survived hospital discharge. Foal survival was not different among delivery methods (*p* = 0.145). However, survival was significantly associated with shorter dystocia duration (*p* < 0.01) and was higher when no obstetrical intervention had been attempted prior to referral (*p* = 0.0436). Among mares, 23.2% did not survive to hospital discharge, most commonly due to endotoxemia or sepsis.

**Conclusion:**

Duration of dystocia prior to hospital admission was a key factor associated with neonatal survival, highlighting the importance of early recognition of dystocia, close monitoring of mares approaching foaling, and prompt referral to specialized equine hospitals.

## 1 Introduction

Dystocia is an uncommon but clinically significant complication in equine reproduction, with reported incidence rates ranging from 2.7 and 10% across different horse populations and study designs (1–4). Overall, approximately 4% of mares experience severe dystocia requiring veterinary intervention (5). However, when obstetrical intervention is not provided promptly, dystocia can result in severe consequences for both mare and foal, including death. The rapid deterioration of fetal viability during equine dystocia is primarily attributed to the relatively early separation of the chorioallantois from the endometrium during the expulsive stage of parturition, leading to acute fetal hypoxia (2, 6). Dystocia may be classified according to maternal, fetal, or placental origin (7, 8). Fetal causes are most frequently reported, with fetal malpresentation of the head, neck and/or extremities documented in 28 to 37.7% of dystocia cases (1–3). By approximately 7 months of gestation, most equine fetuses assume an anterior longitudinal presentation (9, 10). During this developmental period, the fetal hindlimbs typically become encased within one uterine horn, preventing reversion to a posterior presentation and thereby ensuring that approximately 98.9% of foals are delivered in anterior presentation (11). Failure of this physiological positioning process may predispose to dystocia. The expulsive stage of parturition (stage II), which commences with rupture of the chorioallantois, typically lasts 10 to 30 min, with a reported mean duration of 16.7 min and completion within 20 min in 71.7% of parturitions (3). This stage may be prolonged in primiparous mares (12). An extended duration of stage II is associated with heightened occurrence of stillbirths and increased morbidity and mortality in live-born foals (2, 3, 13). Previous studies have demonstrated that each 10-min delay in stage II beyond 30 min increases the risk of stillbirth by 10% and reduces survival to discharge of live-born foals by 16% (13). Reported mare survival rates range from 86% (13) to 91% (7). The critical nature of timing in dystocia management extends beyond the duration of parturition itself to include the time required for veterinary assessment, potential intervention attempts by referring veterinarians, and transport to referral facilities when advanced obstetrical assistance is required. Four generally accepted obstetrical interventions are employed to resolve equine dystocia: (1) assisted vaginal delivery (AVD), in which the conscious mare is manually assisted in vaginal delivery of an intact foal; (2) controlled vaginal delivery (CVD), in which the mare is placed under general anesthesia and manually assisted in vaginal delivery of an intact foal; (3) fetotomy, in which the foal is sectioned and removed vaginally from either a sedated or anesthetized mare; and (4) caesarean section, in which the anesthetized mare undergoes laparotomy for surgical removal of the fetus from the uterus (7, 14, 15). The selection of intervention methods depends on multiple factors including fetal viability, nature of the malpresentation, duration of dystocia, maternal condition, and available resources and facilities. Sweden is one of the most horse-dense countries in Europe (16), with the equine population mainly concentrated in the southern and south-central regions (17). Despite this regional concentration, the country’s large geographical area as well as the uneven distribution of horses and specialized equine referral hospitals often necessitate long transport distances and times before mares with dystocia can receive advanced obstetrical care. Although approximately 13,000 mares are bred annually, most horses are kept for leisure purposes, while only 20% are used commercially, including for racing, riding schools, and boarding activities (16). These demographic, geographical, and management characteristics of the Swedish equine population differ from dominating the existing literature on equine dystocia, which has largely originated from the United States and is based on intensive breeding systems with high densities of stud farms (7, 18). Consequently, the Swedish referral setting provides an opportunity to investigate dystocia in a geographically dispersed and more heterogeneous equine population, where longer referral distances and different management practices may influence the timing of referral, pre-hospital obstetrical interventions, and survival outcomes. By evaluating cases from multiple referral hospitals serving this population, the present study extends existing knowledge andimproves the applicability of dystocia research to equine populations managed outside intensive breeding systems. We hypothesized that prolonged total dystocia duration and delays prior to hospital arrival would be strongly associated with reduced neonatal survival to discharge, whereas maternal survival would remain high despite intervention complexity. Accordingly, the objectives of this study were to: (1) evaluate mare and foal survival rates to discharge, (2) describe the emergency obstetrical interventions performed in a referral setting, and (3) identify key pre-hospital and clinical factors associated with maternal and neonatal mortality.

## 2 Materials and methods

### 2.1 Study design and data collection

A retrospective study was conducted using medical records from three equine referral hospitals in central Sweden. Records from the period January 2006 to December 2022 were reviewed. These hospitals represent the major equine referral centers in central Sweden and were considered representative in terms of specialist care, surgical expertise, and stud veterinary services.

### 2.2 Ethical approval

This was a retrospective study based on analysis of existing clinical records, no new procedures were performed on animals for research purposes. All obstetrical interventions, anesthesia, and euthanasia decisions documented in this study were performed by veterinarians at the participating referral hospitals as part of routine clinical care. The authors did not perform any surgical procedures, administer anesthesia, or perform euthanasia. Formal ethical approval was not required according to Swedish national regulations for retrospective studies utilizing existing anonymized clinical records. All data were handled in accordance with institutional data protection policies and client confidentiality was maintained. All clinical records were anonymized prior to analysis, with client and patient identifiers removed to maintain confidentiality in accordance with institutional data protection policies.

### 2.3 Case selection

Cases were included if mares received emergency obstetrical assistance for dystocia at one of the participating hospitals. Mares already hospitalized for high-risk pregnancy management or other medical conditions were included if they subsequently required assisted delivery due to dystocia. Exclusion criteria were: (1) caesarian section performed during colic surgery; (2) elective caesarean sections (e.g., for mummified fetuses identified during routine gynecological examination); (3) surgical correction of uterine torsion substantially prior to expected parturition; (4) caesarean sections performed for hydrops allantois management; and (5) induced parturitions due to maternal illness, unless induction resulted in dystocia requiring intervention.

### 2.4 Data extraction

When available, the following data were extracted from the medical records: hospital identification;

mare signalment (breed, age); parity (primiparous vs. multiparous); gestation length; involvement of the referring veterinarian (examination and/or attempted assisted delivery); duration of dystocia at hospital arrival (time from chorioallantois rupture to arrival); total duration of dystocia (time from chorioallantois rupture to delivery); duration of obstetrical intervention performed; primary cause of dystocia; type of fetal malpresentation; mare and foal survival; and postpartum complications. Gestation length was categorized as: <300 days, 300–320 days, or >320 days (full-term). Duration of dystocia was recorded in hours from the time of chorioallantois rupture (“water breaking”) to delivery of the foal. Duration of obstetrical intervention at the hospital was recorded in minutes from mare arrival to resolution of dystocia.

### 2.5 Definitions

Dystocia was defined as abnormal or difficult parturition requiring veterinary assistance beyond minimal intervention.

Obstetrical intervention types included:

- Assisted vaginal delivery (AVD): manual correction of fetal malpresentation and/or traction of the standing mare.

- Controlled vaginal delivery (CVD): manual correction and/or traction with the mare under general Anesthesia.

- Fetotomy: trans-vaginal sectioning and removal of a deceased fetus.
- Caesarean section (CS): surgical delivery via ventral midline laparotomy.

Primary cause of dystocia was classified as:

- Fetal: fetal malpresentation (abnormal presentation, position, or posture) or fetal malformation.
- Maternal: uterine torsion at term, uterine inertia, anatomical abnormalities of the birth canal.
- Placental: premature placental separation (red bag delivery) or abortion.

**Figure 1:**
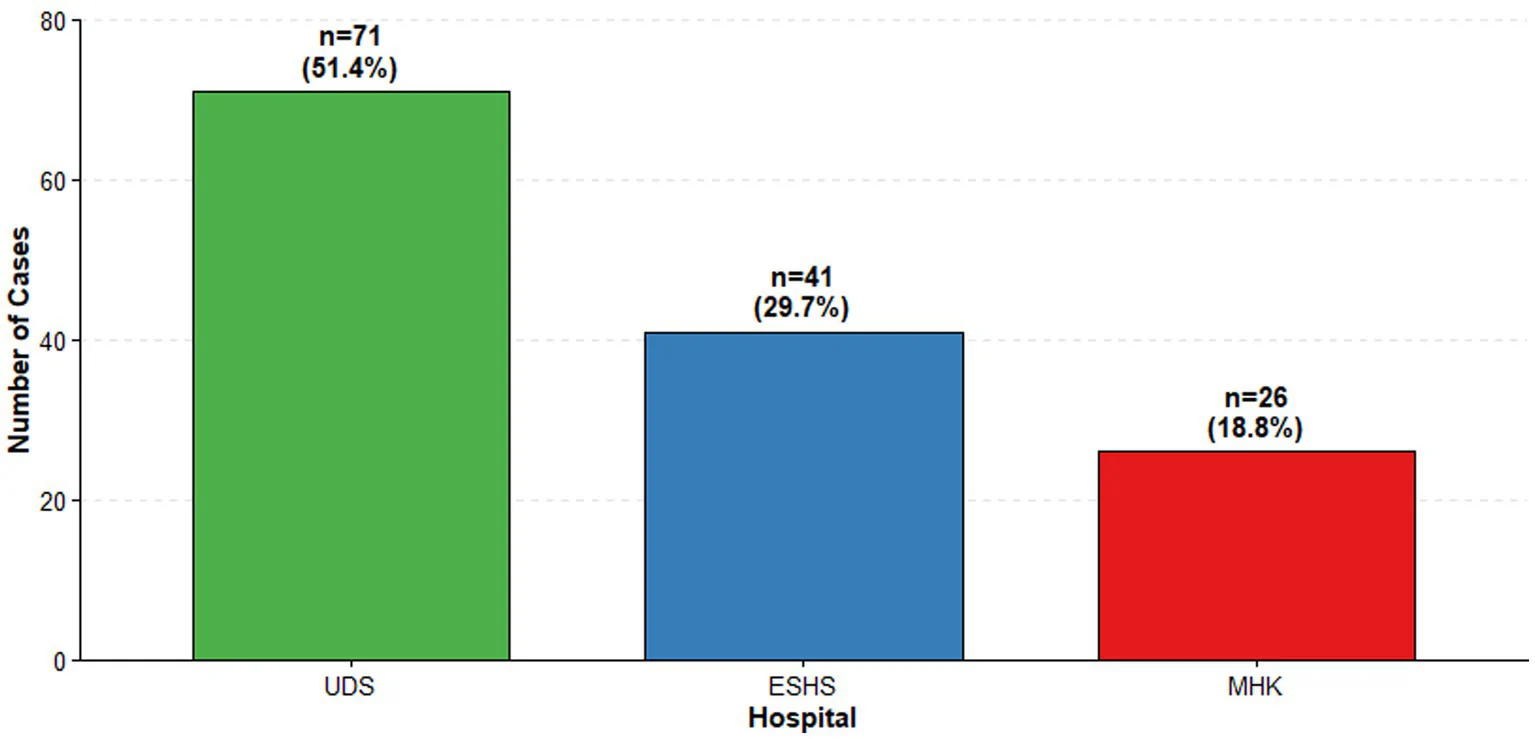
Distribution (%) of equine dystocia cases among three Equine hospitals during the period from January 2006 to December 2022.

Survival was defined as survival to hospital discharge for both mares and foals. Complications were recorded as documented in medical records during hospitalization. Fatal complications were defined as those directly resulting in death or necessitating euthanasia. Cases where euthanasia was performed for non-medical reasons (e.g., financial constraints) were documented separately Figure 1.

### 2.6 Statistical methods

**Figure 2:**
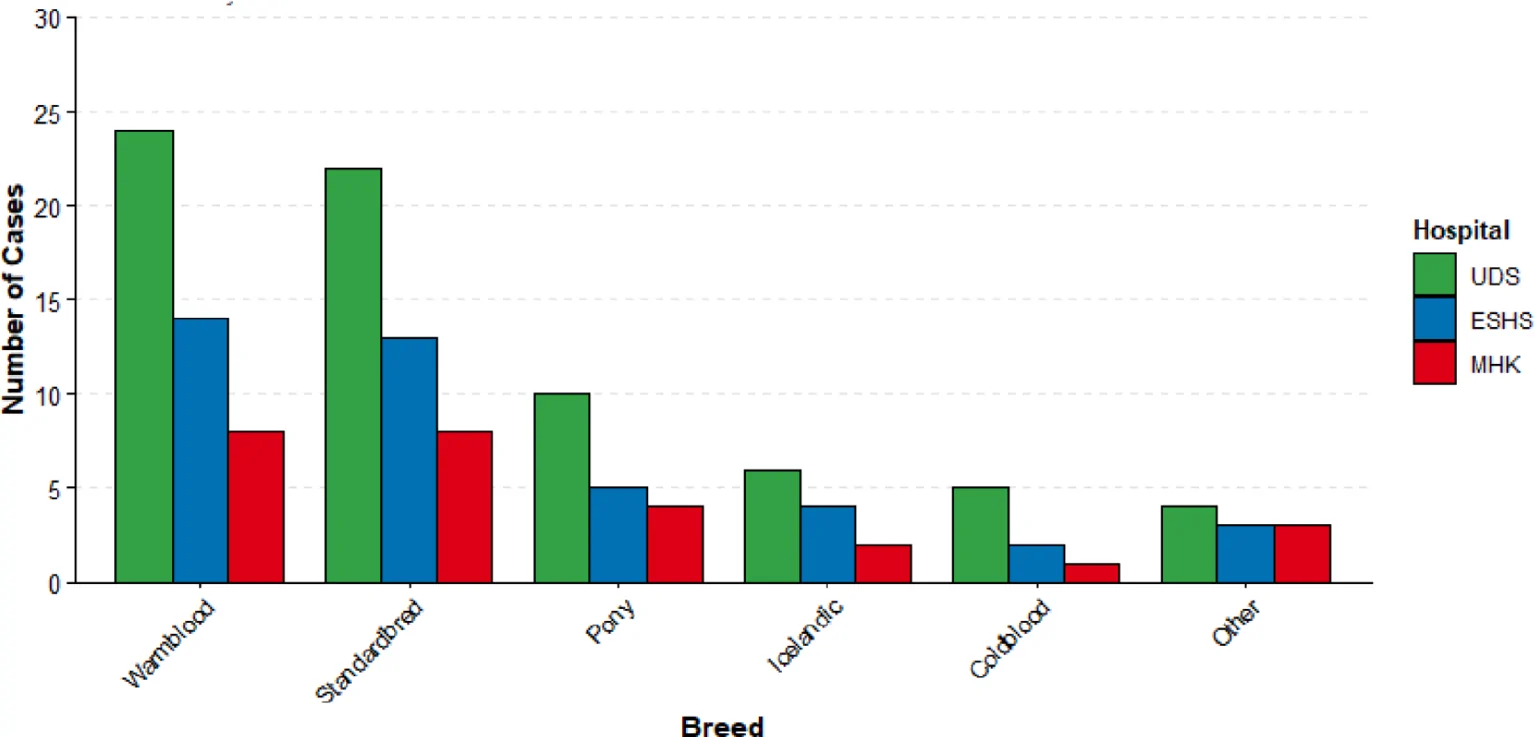
Breed distribution of equine dystocia cases presented to three referral equine hospitals during the period from January 2006 to December 2022.

**Figure 3:**
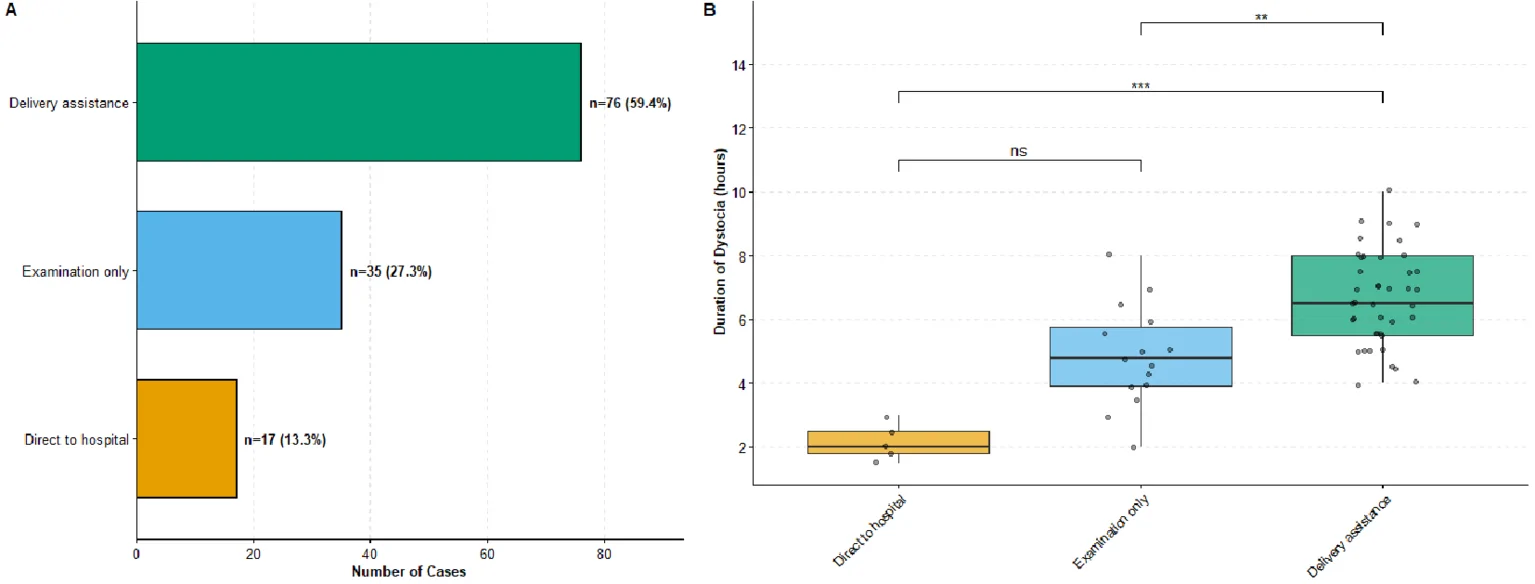
**(A)** Pre-referral equine dystocia management. **(B)** Duration of equine dystocia on hospital admission. NS, Not significant, ** = *p* < 0.01, *** = *p* < 0.001.

**Figure 4:**
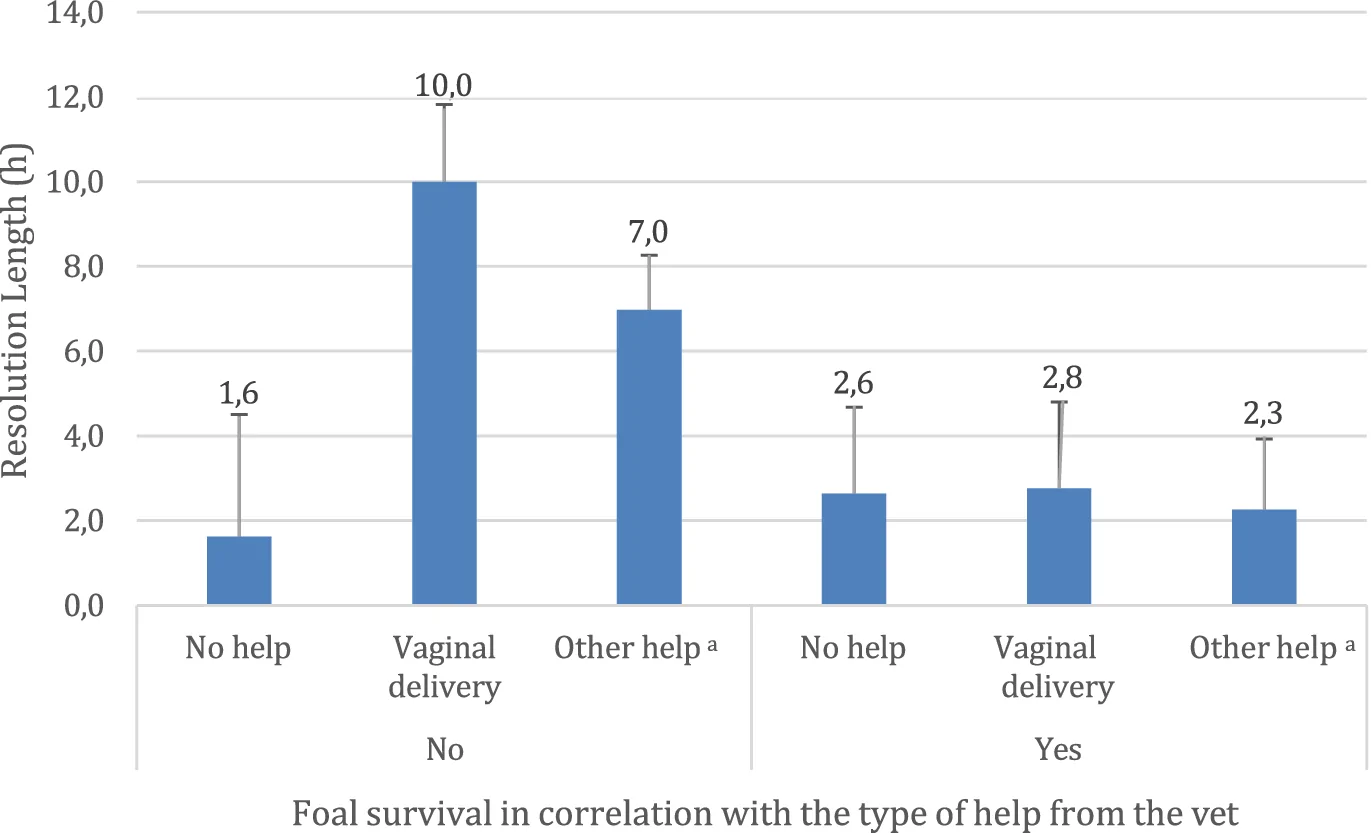
Foal survival in correlation with resolution length (h) and the type of veterinary assistance provided (no help, vaginal delivery, or other help).^**a**^ Other help: Refers to field interventions by the referring veterinarian other than assisted vaginal delivery, such as medication administration (e.g., oxytocin, sedatives, or tocolytics), repositioning, or emergency patient stabilization prior to referral.

Descriptive statistics were calculated using SAS version 9.4 (19). Categorical variables were summarized using frequencies and percentages (*n*, %). Descriptive occurrence estimates are reported using all completed records, while analyses of specific health outcomes were conducted using complete-case data. For continuous variables, descriptive statistics (mean, standard deviation, median, min, and max) were calculated using the MEANS procedure. Binary variables (survival, complications) were evaluated via logistic regression or Fishers’s exact tests. Continuous comparisons across groups were analyzed using General Linear Models via MIXED procedure (PROC MIXED). Least squares means (LSM ± SEM) estimated by the models were adjusted using the Scheffé adjustment for multiple post-ANOVA comparisons and compared. Statistical analyses for categorical outcomes in Figure 2 were performed using the chi-squared (*χ*^2^) test with Monte Carlo simulation. For non-parametric continuous comparisons in Figure 3, group differences were conducted using the Wilcoxon rank-sum test with Bonferroni correction for multiple comparisons, in combination with a Kruskal–Wallis test (*χ*^2^ = 23.29, df = 2). For Figure 4, Fisher’s exact test was applied, followed by *post hoc* pairwise comparisons with Bonferroni correction. Figures were produced in R (version 4.5.1; R Core Team, Vienna, Austria, 2020) within the RStudio platform (version 1.4.1106; RStudio, Boston, Massachusetts, United States, PBC (34)). Statistical significance was set at *p* < 0.05. Multivariable logistic regression was not performed due to low event counts for primary outcomes (*n* = 32 non-surviving mares; *n* = 17 surviving foals), which would violate standard events-per-variable guidelines and lead to model overfitting.

## 3 Results

### 3.1 Case distribution and population characteristics

During the study period, a total of 138 cases met the predefined case definition; of these, 51.4% (*n* = 71) were reported from UDS, 29.7% (*n* = 41) from ESHS, and 18.8% (*n* = 26) from MHK equine hospitals (Figure 1). The distribution of cases among the three veterinary hospitals reflects differences in referral patterns and hospital caseloads during the study period. In association with these cases, 140 foals were delivered, attributable to two twin pregnancies that were first identified upon admission to the hospital. The mean age of the mares was 11.9 years (range: 4–22 years). Age information was unavailable for 10 mares. Mare age did not differ significantly among the three veterinary hospitals (*p* = 0.99).

Warmblood horses constituted 33.3% (*n* = 46) of the study population, Standardbreds represented (31.2%, *n* = 43), ponies (13.8%, *n* = 19; primarily Shetland ponies and Miniature Horses), Icelandic Horses (8.7%, *n* = 12), and draught horses (5.8%, *n* = 8). The remaining 7.2% (*n* = 10) comprised other breeds, including Quarter Horses, Thoroughbreds, Pura Raza Española, and crossbred horses. Breed distribution differed significantly among the veterinary hospitals (*p* = 0.041), with Warmbloods and Standardbreds predominating at UDS, ponies more frequently represented at ESHS, and a more heterogeneous breed distribution observed at MHK (Figure 2). Although breed distribution differed significantly between participating hospitals, potential breed-specific differences in dystocia characteristics or survival outcomes could not be formally evaluated due to small subgroup counts within individual breeds. Parity information was available for 81 mares, of which 33.3% (*n* = 27) were classified as primiparous and 66.7% (*n* = 54) as multiparous. Among the multiparous mares, 20 had had two pregnancies, while 34 had experienced more than two pregnancies. Dystocia had been recorded in at least one previous pregnancy in four mares. Gestation length was documented in 104 cases. Of these, 77.9% (*n* = 81) were full-term (>320 days), 6.7% (*n* = 7) ranged from 300 to 320 days, and 15.4% (*n* = 16) were shorter than 300 days.

The primary causes of dystocia were classified into three categories: maternal, fetal, and placental. Maternal causes accounted for 8 cases of dystocia and included uterine torsion (*n* = 3), uterine inertia (*n* = 3), prepubic tendon rupture (*n* = 1), and incomplete dilatation of the birth canal (*n* = 1). Fetal causes were the most frequently identified, comprising 114 cases. Malpresentation was the predominant fetal cause (*n* = 106), followed by fetal malformations (*n* = 5) and twin pregnancies (*n* = 3). When cases of abortion in which malposition contributed to dystocia were included, malposition accounted for approximately 86% of all dystocia cases observed in this study. Placental causes were identified in 16 cases and consisted primarily of abortion (*n* = 15), with one case of red bag delivery (premature separation of the chorioallantois; *n* = 1).

### 3.2 Pre-hospital interventions

Pre-hospital management was documented in 92.8% (128/138) of cases (Figure 3). Among these, 59.4% (76/128) received obstetrical intervention by the referring veterinarian prior to hospital arrival, including assisted vaginal delivery, controlled vaginal delivery, or fetotomy. In 27.3% (33/128) of cases, the referring veterinarian performed vaginal examination but did not attempt delivery assistance, usually because the fetal malpresentation was deemed too complex or time-consuming to resolve on-farm. The remaining 13.3% (17/128) were transported directly to the hospital without prior veterinary assessment.

Duration of dystocia at hospital arrival, defined as the time elapsed from chorioallantoic rupture to arrival, was recorded in 44.2% (61/138) of cases. Median duration was 6.5 h (IQR: 5.0–7.5 h; range: 1.5–10.0 h) and differed significantly among pre-hospital management groups (*p* < 0.001). Mares transported directly to the hospital had the shortest median duration (2.0 h; IQR: 1.8–2.5 h), followed by those examined only (4.8 h; IQR: 3.9–5.75 h), while mares receiving pre-hospital delivery assistance had the longest duration (6.5 h; IQR: 5.5–8.0 h).

Post-hoc pairwise comparisons revealed significant differences between all groups (direct vs. examination only: *p* = 0.009; direct vs. delivery assistance: *p* < 0.001; examination only vs. delivery assistance: *p* = 0.002). In 16.7% (23/138) of cases, parturition was unmonitored and dystocia was only detected after an unknown duration of time had elapsed.

### 3.3 Hospital-based interventions

Dystocia was successfully resolved in 96.4% (133/138) of cases through obstetrical intervention. Euthanasia was performed in 4.3% (6/138) of cases, either due to complications precluding successful intervention (*n* = 4) or intraoperatively during caesarean section (*n* = 2), with decisions based on medical prognosis or owner request.

**Figure 5:**
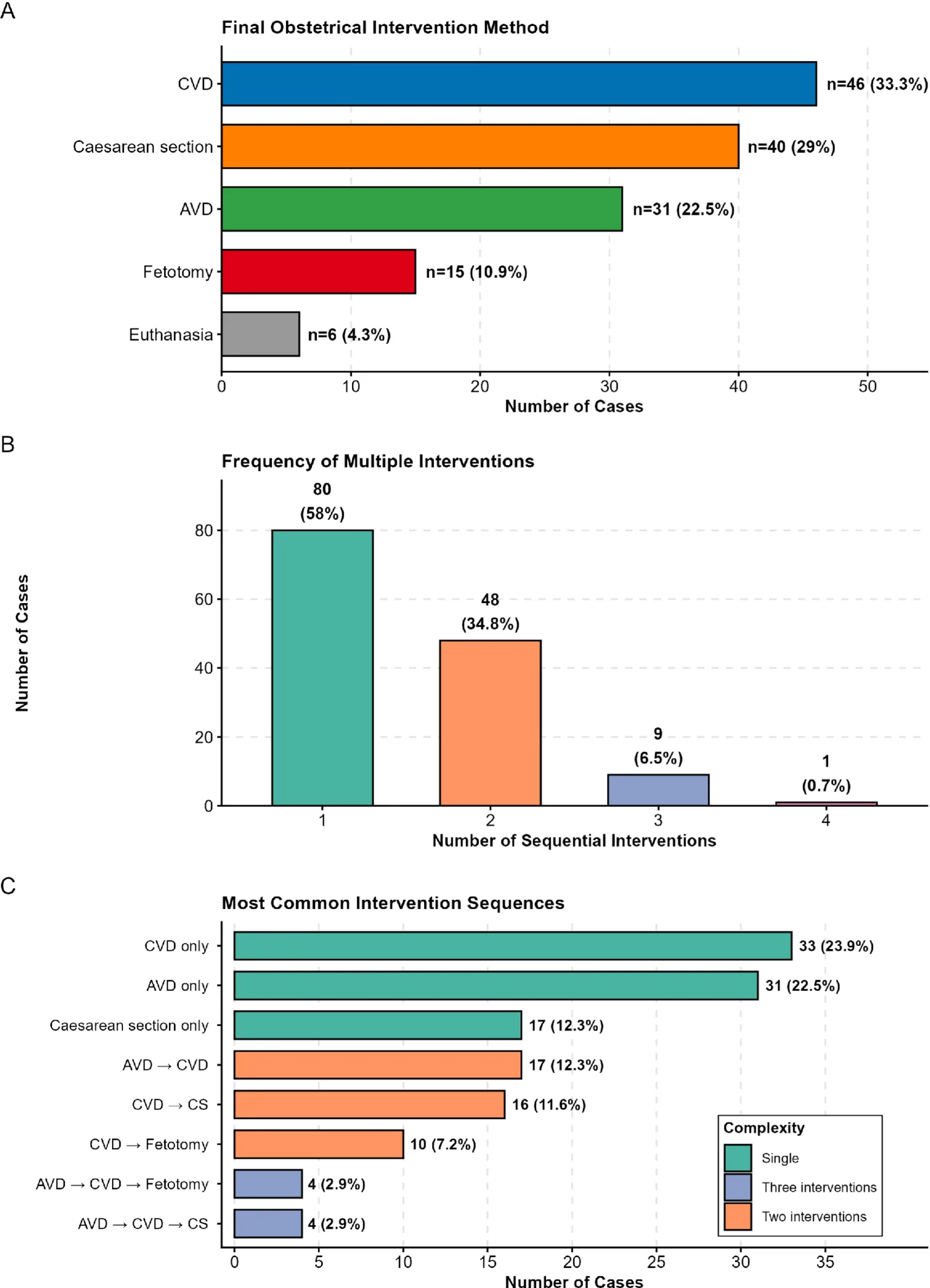
Distribution of obstetrical interventions used to resolve equine dystocia. **(A)** Final intervention used for dystocia resolution. **(B)** Number of sequential interventions performed per case. **(C)** Most common intervention sequences used for dystocia resolution. AVD, assisted vaginal delivery (manual correction of fetal malpresentation and/or traction in the standing mare); CVD, controlled vaginal delivery (manual correction and/or traction with the mare under general anesthesia); Fetotomy (trans-vaginal sectioning and removal of a deceased fetus); CS, caesarean section (surgical delivery via ventral midline laparotomy).

The distribution of obstetrical interventions employed is presented in Figure 5 (panel A). Controlled vaginal delivery (CVD) was the most frequently utilized method (33.3%, 46/138), followed by caesarean section (29.0%, 40/138), assisted vaginal delivery (AVD; 22.5%, 31/138), and fetotomy (10.9%, 15/138). In 58.0% (80/138) of cases, dystocia was resolved with a single intervention method. Multiple sequential interventions were required in 42.0% (58/138) of cases, with two.

Methods employed in 34.8% (48/138), three methods in 6.5% (9/138), and four methods in 0.7%.

(1/138). The most common sequential intervention patterns were: CVD followed by caesarean.

Section (11.6%, 16/138), AVD followed by CVD (12.3%, 17/138), and CVD followed by fetotomy.

(7.2%, 10/138). Terminal caesarean sections (predetermined euthanasia during surgery) were performed in 1.4% (2/138) of cases.

### 3.4 Foal survival and assciated factors

#### 3.4.1 Overall foal survival

Of the 140 foals delivered, 17.1% (24/140) were born alive and 12.1% (17/140) survived to hospital discharge. Among the 7 foals that died or were euthanized after live birth, causes included prematurity or dysmaturity (*n* = 5, 71.4%), patellar luxation (*n* = 1, 14.3%) and suspected anaphylactic reaction to intravenous plasma administration (*n* = 1, 14.3%). All foals surviving to discharge were delivered at full-term gestation (>320 days; *n* = 81/81), whereas no foal survival was observed in foals delivered before 320 days of gestation (0%, *n* = 0/23; including *n* = 7 delivered at 300–320 days and *n* = 16 delivered at < 300 days).

Foal survival rates varied numerically among delivery methods but did not differ significantly (*p* = 0.145). Survival was highest following AVD (8/31, 25.8%), followed by CVD (5/46, 10.9%), and caesarean section (4/40, 10.0%). Fetotomy had a 0% survival rate (0/15) because it was performed solely on deceased foals. No significant differences were identified among the delivery methods (*p* > 0.05).

Among the 17 foals surviving to discharge, the primary cause of dystocia was fetal malposition (14/17, 82.4%), maternal uterine inertia (2/17, 11.8%), and premature placental separation (1/17, 5.9%). Malposition predominantly involved abnormal head and/or neck positioning (9/14, 64.3%).

#### 3.4.2 Impact of dystocia duration on foal survival

**Figure 6:**
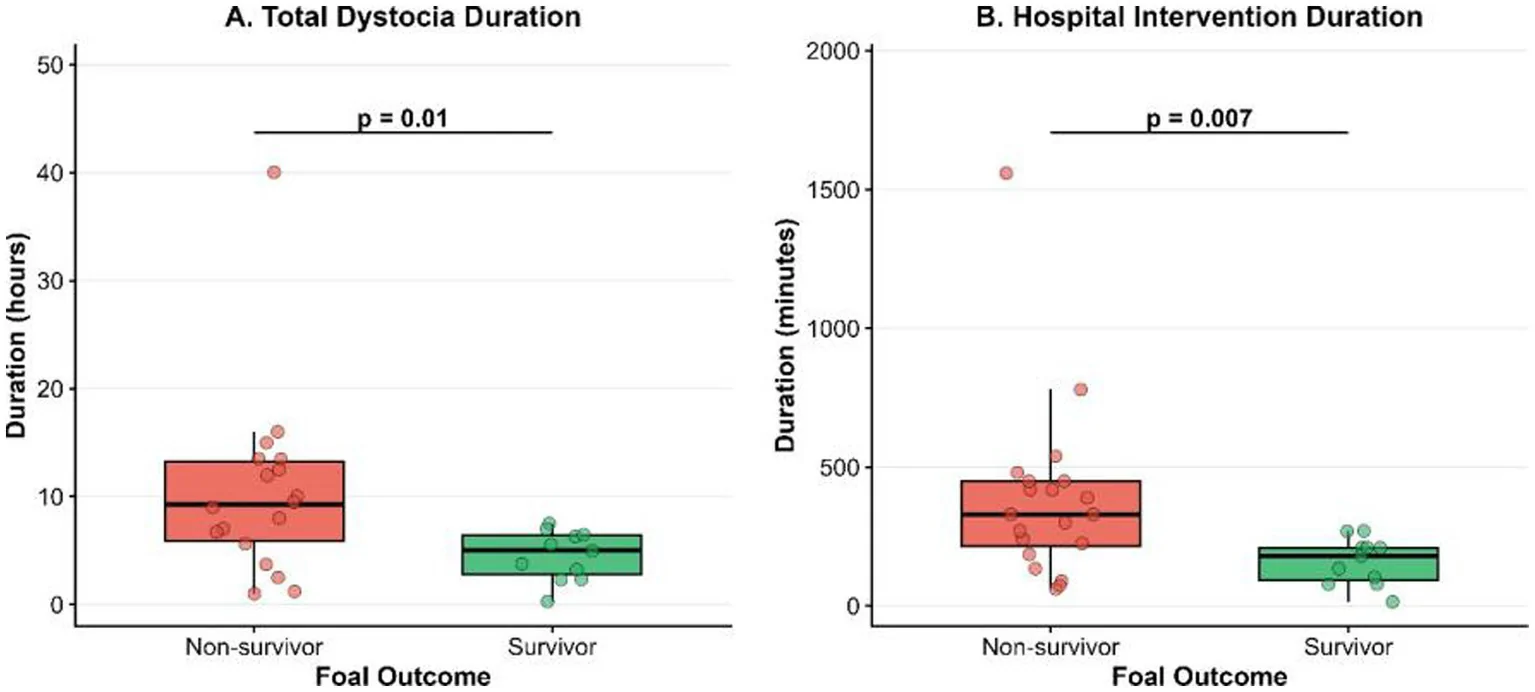
Association between duration and foal survival. **(A)** Effect of total dystocia duration on foal survival (*n* = 30). **(B)** Effect of duration of in-hospital obstetrical interventions on foal survival (*n* = 31).

Foal survival was associated with a significantly shorter total dystocia duration (*p* < 0.01; survivors: media 5 h, non-survivors: median 9.5 h). The duration of obstetrical assistance provided at the equine hospital was significantly shorter for survivors compared to non-survivors (*p* = 0.007; survivors: median 180 min, non-survivors: median 330 min), independent of the intervention applied to resolve the dystocia (Figure 6).

## 4 Resource identification initiative

### 4.1 Impact of pre-hospital intervention on foal survival

Foal survival in relation to obstetrical interventions performed by the referring veterinarian prior to admission demonstrated was improved (*p* = 0.0436) when no medical intervention had been administered before presentation to the clinic (Figure 4).

### 4.2 Mare survival and complications

#### 4.2.1 Overall mare survival

**Figure 7:**
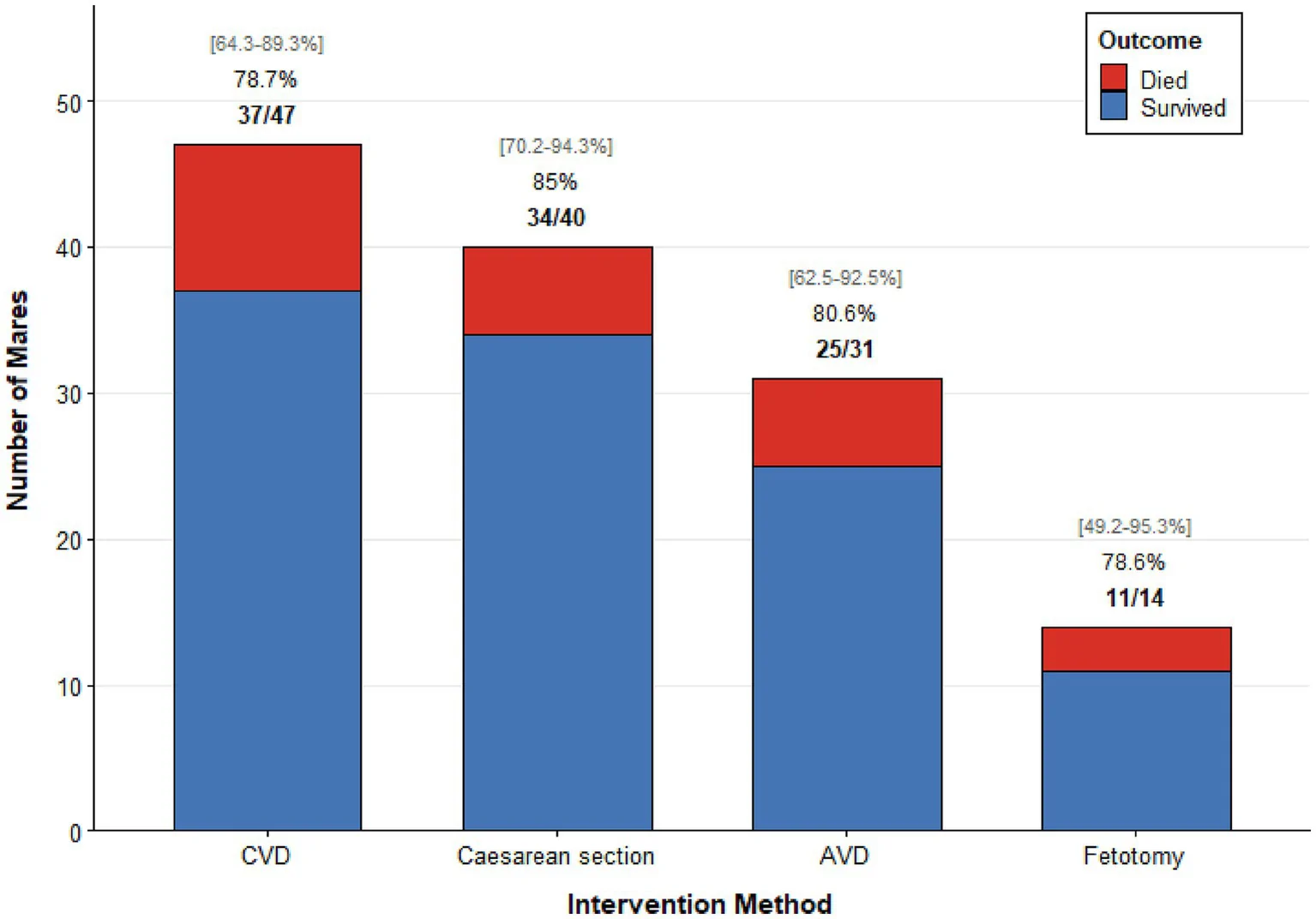
Mare survival to hospital discharge by intervention method. Stacked bars represent the number of mares that survived (blue) or died (red) to hospital discharge for each intervention method. Numbers above each bar indicate the number of surviving mares out of the total, followed by the survival percentage and 95% confidence interval (CI) in brackets. No significant difference in survival to discharge was identified among intervention CVD, controlled vaginal delivery; AVD, assisted vaginal delivery.

Of the 138 mares referred for dystocia, 4 were euthanized and 2 underwent terminal caesarean section and were excluded from further analysis. Of the remaining 132 mares, 128 (97.0%) survived parturition. In total, 107 mares (81.1%) survived to hospital discharge (see Figure 7 for survival rate per intervention). The latter group included 14 mares that were discharged directly after delivery and managed with continued care at home. No significant association was identified between mare survival and the duration of dystocia at the time of hospital admission.

#### 4.2.2 Fatal complications

**Figure 8:**
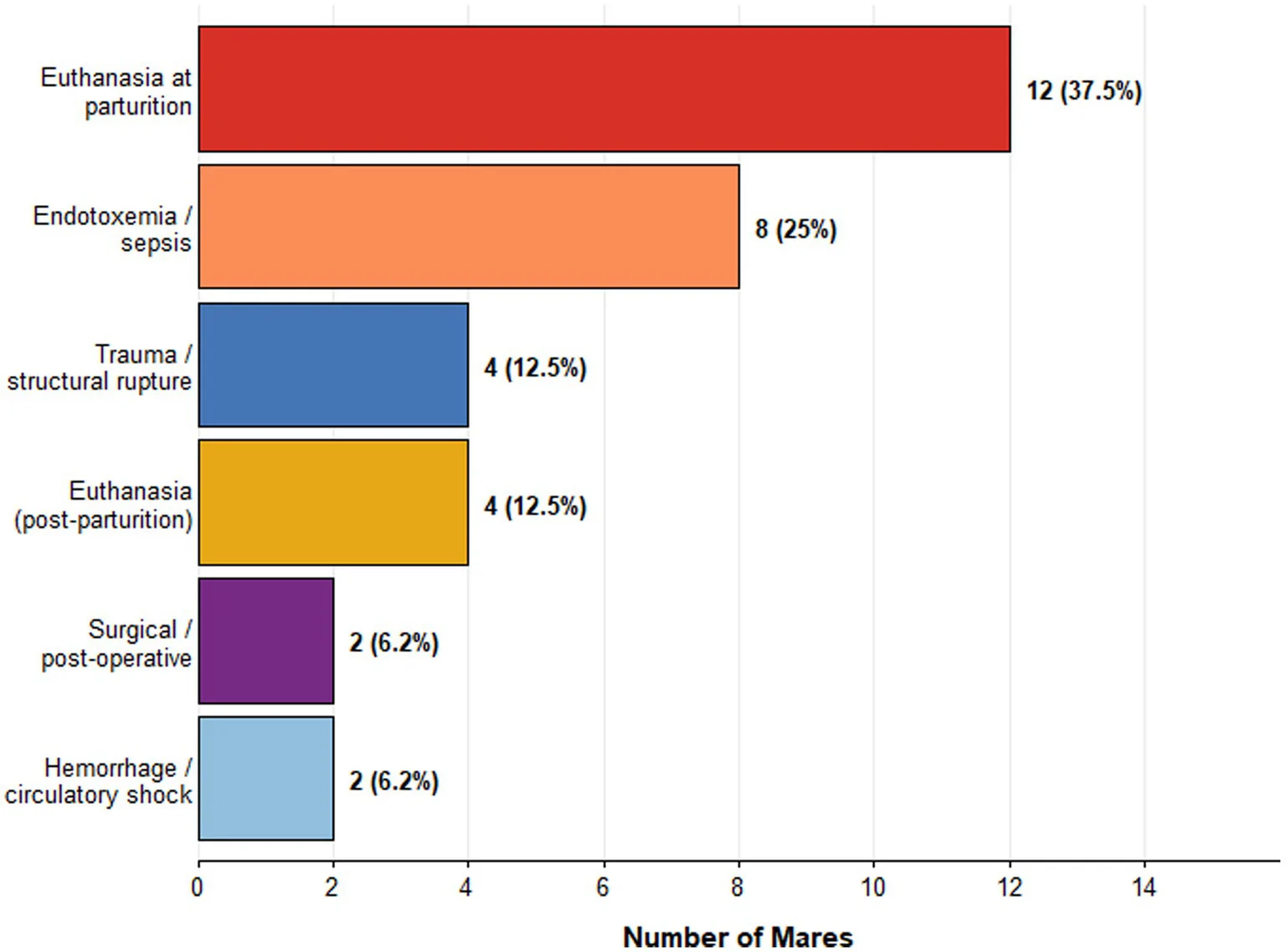
Distribution of causes of death or euthanasia in mares referred for dystocia that did not survive to hospital discharge (*n* = 32). Causes included complications directly associated with dystocia, obstetrical interventions, and secondary systemic or reproductive disorders identified during hospitalization.

Among the 138 mares referred for dystocia, 32 (23.2%) did not survive to hospital discharge. Twelve mares (12/32, 37.5%) were euthanized during or immediately after parturition due to severe dystocia-related complications deemed incompatible with life. Among the remaining 20 mares, endotoxemia or sepsis was the most common cause of death or euthanasia (8/32, 25.0%), followed by trauma or structural rupture (4/32, 12.5%) and euthanasia for post-parturition complications (4/32, 12.5%). Hemorrhage or circulatory shock and surgical or post-operative complications each accounted for 2 cases (2/32, 6.2%) (Figure 8).

#### 4.2.3 Risk factors for Mare and Foal survival

Continuous and categorical predictors of survival outcomes were evaluated using univariable logistic regression (Table 1). Dystocia duration prior to hospital arrival was significantly associated with decreased foal survival to discharge (OR = 0.56, 95% CI: 0.38–0.83, *p* = 0.0035), representing a 43.8% reduction in the odds of foal survival for each additional hour of delay. Similarly, foals from mares that underwent attempted obstetrical intervention by a referring veterinarian prior to transport had lower odds of survival to discharge (OR = 0.26, 95% CI: 0.08–0.89, *p* = 0.0316).

**Table 1:** Univariable logistic regression analysis for mare survival to discharge and foal survival to discharge.

Predictor variable	Category/unit	Mare survival at referral clinic (OR)	95% CI	*p*-value	Foal survival to discharge (OR)	95% CI	*p*-value
Dystocia reason	Reason 1*** (Ref)	1.00	—	—	1.00	—	—
	Reason 2***	1.89	0.21–17.34	0.572	0.4	0.08–2.29	0.316
	Reason 3***	>999.999*	<0.001- > 999.99*	0.961	0.2	0.02 – 2.65	0.222
Referral vet intervention	No (Ref)	1.00	—	—	1.00	—	—
	Yes	1.98	0.38–10.44	0.420	0.26	0.08–0.89	0.032
Fetotomy Performed	No (Ref)	1.00	—	—	—	—	—
	Yes	1.38	1.16–11-68	0.767	—**	—**	—**
Dystocia Duration	Per 1-h increase	0.98	0.77–1.25	0.891	0.56	0.38–0.83	0.003

*Odds ratio and confidence interval were non-estimable (>999.99) due to quasi-complete separation resulting from 100% mare survival (0% mortality) within the reason 3 subgroup.

**Estimates could not be generated due to complete separation (0% foal survival in cases requiring fetotomy).

*** Reason 1 (Ref): Fetal Malposition/Malpresentation; Reason 2: Maternal and Uterine/Cervical Factors (Uterine torsion, Cervical stenosis, Uterine inertia, etc.); Reason 3: Fetal Factors and Abnormalities (Fetal oversize, Abortion/Aborted fetus, Twins, Malformations).

In contrast, mare survival to discharge was not significantly influenced by dystocia duration (OR = 0.98, 95%, CI: 0.77–1.25, *p* = 0.891), prior referral veterinarian intervention (OR = 1.98, 95%, CI: 0.38–10.44, *p* = 0.420), or performance of a fetotomy (OR = 1.38, 95%, CI: 0.16–11.68, *p* = 0.767).

## 5 Discussion

### 5.1 Study population and comparison with literature

The existing literature on equine dystocia and foal survival is dominated by studies originating from the United States (7, 18), where high densities of stud farms and intensive breeding management systems prevail. While these studies have contributed substantially to current understanding, their applicability to other equine populations may be limited. In Sweden, the equine population may be more geographically dispersed than in the United States, and breeding and management practices could differ considerably, potentially influencing both the occurrence and management of dystocia. Furthermore, many previous investigations have focused on a restricted number of breeds, most commonly Standardbreds (2), Thoroughbreds (7), or combinations of two breeds (18), which further limits extrapolation of reported outcomes to more heterogeneous populations. In contrast, the present study included mares from nine distinct breeds in addition to several crossbreeds, thereby reflecting a broader and more representative breeding population. Nevertheless, the limited number of parturitions within individual breeds and the unequal distribution of breeds across the three participating referral hospitals precluded meaningful statistical assessment of breed-specific predispositions to dystocia. This limitation underscores the need for larger, multicenter studies to better evaluate potential breed-related risk factors in diverse equine populations.

### 5.2 Causes of dystocia

Maternal causes of dystocia were infrequently observed in the present study, accounting for 5.8% (8/138) of cases. This finding is partially consistent with previous literature, in which primary uterine inertia and incomplete dilation of the birth canal have been reported as the most common maternal causes (2, 3, 12, 14, 33). In contrast, uterine torsion (37.5%, 3/8) and uterine inertia (37.5%, 3/8) were the predominant maternal causes identified in this study. However, given the small number of maternal cases, these differences should be interpreted with caution.

### 5.3 Pre-hospital management and referral timing

In the present study, more than half of the mares (59.4%; 76/128) received one or more obstetrical interventions by the referring veterinarian prior to hospital admission, including attempted assisted vaginal delivery, controlled vaginal delivery, or fetotomy, without successful resolution of the dystocia. This high prevalence underscores the substantial reliance on on-farm obstetrical management in equine dystocia prior to referral. Stage II of normal parturition in the mare is physiologically brief and typically completed within 20–30 min (20). Any prolongation of this phase represents a critical obstetrical emergency, as premature separation of the chorioallantois can rapidly compromise placental oxygen exchange and precipitate acute fetal hypoxia (2, 6). In this context, the significantly longer duration of dystocia observed in mares that received pre-hospital obstetrical assistance is particularly notable. These findings show that repeated or prolonged attempts to resolve dystocia under field conditions the likelihood of favorable neonatal outcomes, possibly by inadvertently extending the duration of fetal hypoxia.

Several factors may explain the observed association between pre-hospital intervention and increased dystocia duration. The severity and complexity of the dystocia may have influenced both the decision to attempt field intervention before referral and the subsequent outcome. Consequently, the observed association between pre-hospital intervention and foal survival should be interpreted with caution, as case severity is likely to have acted as a confounding factor. In addition, the experience and skill level of the ambulatory veterinarian may affect the efficiency and outcome of obstetrical maneuvers. Logistical considerations, including the distance between the farm and the referral hospital, as well as financial constraints, may further encourage prolonged on-farm management in an effort to avoid transport or referral costs. Collectively, these factors may delay referral and extend the duration of stage II.

### 5.4 Intervention methods

Controlled vaginal delivery (CVD) was the most frequently utilized method for dystocia resolution in the present study (33.3%, 46/138), followed by caesarean section (29.0%, 40/138), assisted vaginal delivery (AVD; 22.5%, 31/138), and fetotomy (10.9%, 15/138). These findings are comparable to those reported by Freeman et al. (21), who documented CVD use in 29% of cases, largely due to prolonged second-stage labour associated with vaginal edema and desiccation of the vestibulovaginal mucosa, rendering caesarean section the safer alternative. In contrast, other authors have reported CVD as the most commonly employed approach, accounting for approximately 71–85% of dystocia cases, with reported success rates of around 80% (18) and 94% (7). Assisted vaginal delivery is infrequently attempted in equine dystocia (7) and remains the most frequently attempted and preferred first-line method in field settings. However, Woodford (22) described the implementation of a strict two-minute time frame for AVD, enabling rapid progression to preparation for CVD when adequate progress is not achieved. In cases of acute dystocia, a duration of 30 min for CVD may be considered excessive (23). Despite this, CVD offers several advantages, and when performed promptly, rapid progression and successful resolution are often achievable (22). Fetotomy was less frequently performed in the present study, although favorable success rates have been reported in the literature (24, 25). The limited use of fetotomy in equine practice may be attributable to reduced availability of training compared with CVD or caesarean section (22). Given the technical demands of the procedure, a high level of operator skill is essential to minimize trauma to the reproductive tract, particularly the cervix, as such injuries may have serious implications for future fertility (26).

### 5.5 Foal survival determinants

Foal mortality rates reported in previous studies were substantially lower than those observed in the present study, with rates of 20.0% (4/20) reported by Ellerbrock et al. (27) and 21.0% (12/57) by Lanci et al. (2). In both studies, obstetrical assistance was provided on-site by specialized veterinarians, and the mares were either already present at the referral clinic or located in close proximity to it. In contrast, the present study revealed markedly higher foal mortality rates of 82.9% (116/140) immediately after parturition and 87.9% (123/140) at discharge. More comparable outcomes were reported by Freeman et al. (21), who described foal mortality rates of 89.2% (91/102) post-parturition and 95.0% (97/102) at discharge following both surgical and conservative obstetrical interventions. The authors attributed these high mortality rates, at least in part, to prolonged transportation times between the stable and the equine referral hospital. However, when considering the median dystocia duration of 6.5 h at the time of hospital admission observed in the present study, delayed referral associated with transport logistics may still represent an important contributing factor. In particular, the geographic context of Sweden, characterized by large distances between equine facilities and referral centers, may result in prolonged transport times that further extend the duration of dystocia before advanced obstetrical care can be initiated. Consequently, even when veterinary assistance is available in the field, geographical constraints may significantly influence the timeliness of referrals and ultimately affect neonatal survival.

Nevertheless, direct comparisons between these studies should be interpreted with caution. Referral hospitals inherently receive a disproportionate number of severe and complicated dystocia cases, introducing a substantial case selection bias. As previously emphasized by Vandeplassche (28), dystocia managed in referral settings represents a fundamentally different clinical population from cases resolved in routine ambulatory veterinary practice or those occurring in specialized foaling centers. Consequently, outcome data derived from referral clinics are likely to reflect the greater complexity and severity of cases presented, rather than the overall prognosis of equine dystocia in the general population.

Furthermore, the observed negative association between pre-hospital obstetrical intervention by referring veterinarians and foal survival must be interpreted with caution due to potential confounding by indication. It is highly likely that cases undergoing field intervention represented inherently more severe, prolonged, or complicated dystocias, which prompted veterinary assistance in the first place. Thus, the lower foal survival rate in this subgroup may reflect underlying case complexity and cumulative time delays rather than an adverse effect of the field intervention itself.

Foal mortality rates reported in previous studies were substantially lower than those observed in the present study, with mortality rates of 20.0% (4/20) reported by Ellerbrock et al. (27) and 21.0% (12/57) by Lanci et al. (2). In both studies, obstetrical assistance was provided on-site by specialized veterinarians, and the mares were either already present at the referral clinic or located in close proximity to it. In contrast, foal mortality in the present study was 82.9% (116/140) immediately after parturition and 87.9% (123/140) at discharge. Comparable outcomes were reported by Freeman et al. (21), who observed foal mortality rates of 89.2% (91/102) following parturition and 95.0% (97/102) at hospital discharge in a referral population managed using both surgical and conservative obstetrical interventions. Collectively, these findings suggest that referral populations represent a substantially different case mix than mares managed in specialized foaling centres or intensive breeding systems. Although prolonged dystocia has consistently been identified as a major determinant of foal survival (7, 13, 29), none of the previous referral studies, including those of Freeman et al. (21), Lanci et al. (2), and Ellerbrock et al. (27), specifically quantified the duration of dystocia before hospital admission. Likewise, Byron et al. (7) and Norton et al. (13) reported the total duration of Stage II labour and the interval from hospital admission to resolution of dystocia but did not separately evaluate the pre-admission period. Consequently, the influence of referral delay before arrival at a specialist hospital has remained largely unexplored.

In the present study, the median dystocia duration before hospital admission was 6.5 h, and prolonged dystocia before referral was significantly associated with reduced foal survival. In contrast, no association was identified between foal survival and the interval from hospital admission to resolution of dystocia, consistent with the findings of Norton et al. (13), who similarly demonstrated that reducing the time from hospital admission to delivery did not improve foal survival because the total duration of Stage II labour remained unchanged. Together, these findings suggest that factors preceding referral, rather than in-hospital management alone, are the principal determinants of neonatal survival outcome. The prolonged pre-admission dystocia duration observed in the present study may, at least in part, reflect the geographical characteristics of Sweden, where considerable distances between equine facilities and referral hospitals may delay access to specialized obstetrical care. However, owing to the retrospective nature of the study, transport time and distance were not available and therefore could not be evaluated directly. In addition to transport logistics, delayed recognition of dystocia, prolonged attempts at field management, and delayed referral are also likely to have contributed to the prolonged dystocia duration.

Early recognition of dystocia, continuous monitoring by experienced personnel familiar with normal parturition in mares (30), and prompt referral to an equine referral hospital therefore remain critical to improving foal survival. This is further supported by the finding that 16.7% of dystocia cases in the present study were unmonitored, resulting in an unknown duration of dystocia and highlighting an important opportunity for improving on-farm surveillance and timely intervention. Finally, comparisons between studies should be interpreted with caution, as referral hospitals inherently receive a disproportionate number of severe and complicated dystocia cases. As emphasized by Vandeplassche (28), dystocia managed in referral hospitals represents a fundamentally different clinical population from cases managed in ambulatory practice or specialized foaling centres. Consequently, survival rates reported from referral hospitals are likely to reflect the complexity and severity of referred cases rather than the overall prognosis of equine dystocia in the general population.

### 5.6 Mare outcomes

Mare mortality in the present study was 3.0% (4/132) in the immediate post-parturient period and increased to 18.9% (25/132) by the time of hospital discharge. These findings are broadly consistent with those reported by Freeman et al. (21), who documented a mortality rate of 19.8% (23/116). In contrast, lower mare-mortality rates were reported by Lanci et al. (2) and (1), at 8.8% (5/57) and 5% (1/20), respectively. However, both studies were characterized by smaller sample sizes and included mares that were already hospitalized at the time of dystocia, which may have influenced survival outcomes, as discussed previously. In the present study, no association was identified between the duration of dystocia and mare survival, in contrast to the findings observed for foals. This lack of correlation is consistent with previous reports, including Freeman et al. (21). Fatal complications in mares were most commonly attributed to endotoxemia or sepsis (25.0%, 8/32), followed by trauma or structural rupture (12.5%, 4/32). These findings align with the complications most frequently described in the existing literature (31).

### 5.7 Study limitations

The retrospective nature of this study, based on medical records collected from three equine referral hospitals over a 16-year period, introduces inherent limitations. Data were recorded by multiple clinicians, which may have resulted in variability in clinical assessment, case documentation, and interpretation. Furthermore, differences in referral patterns among the participating hospitals may have influenced the case mix, severity of presentation, and clinical outcomes. Information regarding the circumstances preceding referral, such as whether mares were managed on breeding farms with resident veterinarians or whether veterinary assistance was sought only after the onset of dystocia, was not consistently documented and therefore could not be evaluated. As these factors may have influenced the time to intervention and, consequently, survival outcomes, they should be considered when interpreting the findings. In addition, although the present study identified factors associated with foal survival, its retrospective design and the relatively limited sample size within several subgroups precluded the development and validation of multivariable prognostic or predictive models. Future prospective studies including larger case numbers are warranted to develop robust prognostic models that may further support clinical decision-making in both field and referral settings.

The extended study period also introduces the possibility that temporal changes in clinical practice may have influenced case management and outcomes. Advances in evidence-based veterinary medicine have improved clinical decision-making over time (35), while evolving diagnostic and therapeutic approaches may have affected survival independently of individual case characteristics. In parallel, owners have demonstrated increasing utilization of and expenditure on veterinary care (36), suggesting a greater willingness to pursue advanced diagnostics and treatment. Although this trend has primarily been described in companion animals, it may also be relevant to the equine industry, where horses are increasingly regarded as companion animals or family members, potentially influencing treatment decisions and referral practices. Nevertheless, the findings of Norton et al. (13) indicate that improvements in hospital protocols alone are unlikely to compensate for delays occurring before referral. Although organized hospital protocols may increase the efficiency of in-hospital management, prolonged Stage II labour before admission remains the principal determinant of foal survival. Consequently, while advances in veterinary medicine and changes in owner decision-making may have influenced treatment strategies during the study period, timely recognition of dystocia and prompt referral to a specialized equine hospital remain the most critical factors for improving neonatal survival. These potential sources of bias should be considered when interpreting the findings and their applicability to broader equine populations.

### 5.8 Clinical implications

This study demonstrated that fetal causes of dystocia were substantially more frequent than maternal causes. This finding is consistent with previous reports indicating that equine dystocia is predominantly of fetal origin in both retrospective studies (1, 2, 8) and prospective investigations (27). In agreement with the existing literature, abnormal fetal positioning or posturing was identified as the most frequently diagnosed cause of dystocia (5, 32).

## 6 Conclusion

This study provides novel insight into the clinical characteristics of equine dystocia and associated foal survival within a Swedish referral population. The duration of dystocia prior to arrival at the referral hospital was identified as a key factor associated with neonatal survival, highlighting the profound impact of time on fetal viability during equine parturition. These findings emphasize the importance of vigilant periparturient management. Early recognition of dystocia, facilitated by close monitoring of mares approaching their expected foaling date, is essential to allow timely intervention. Furthermore, the results support a management approach that prioritizes rapid referral to a facility equipped for advanced obstetrical care rather than prolonged attempts at on-farm resolution.

Taken together, minimizing delays between the onset of dystocia and definitive treatment appears crucial for optimizing neonatal outcomes. Increased awareness among breeders and veterinarians regarding the time-critical nature of equine dystocia may therefore represent an important step toward improving foal survival.

## Data Availability

The datasets presented in this study can be found in online repositories. The names of the repository/repositories and accession number(s) can be found in the article/supplementary material.
